# Supplementary material for: Inhibition of Glutamate Release from Rat Cortical Nerve Terminals by Dehydrocorydaline, an Alkaloid from Corydalis yanhusuo
Source: Molecules. 2022 Jan 31;27(3):960. doi: 10.3390/molecules27030960 (PMC8838318; doi:10.3390/molecules27030960)
Supplement: Supplementary file 1 [file molecules-27-00960-s001.zip › molecules-1556292-supplementary.pdf]

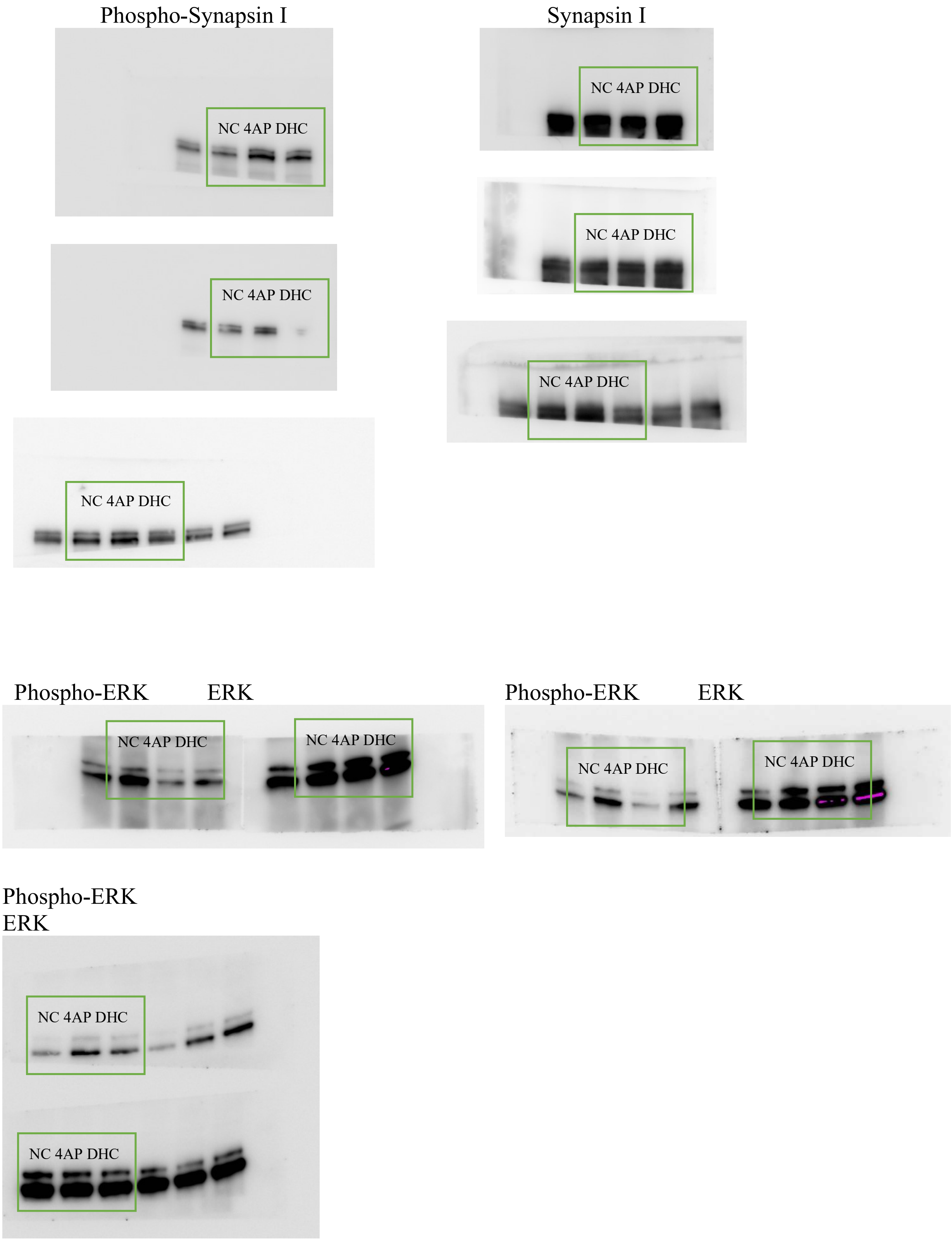

Figure S1. Original images of Western Blot.

Analysis Report

<Sample Information>

Sample Name : CFN90330
Instrument : SHIMADZU LC-20AT
Column : Wonda Cract ODS-2 (5 μm, 4.6×250 mm)
Column Temp. : 35°C
Mobile phase flow : 1.0 ml/min
Injection Volume : 5 uL
Detection Wave. : UV-276nm
Con. & Solvent : 0.5mg/ml (Methanol)

<Analysis condition>

5%→100% Acetonitrile in 0.05% Phosphoric acid H2O
over 10.0min followed by 100% Acetonitrile to 17.0 min

<Chromatogram>

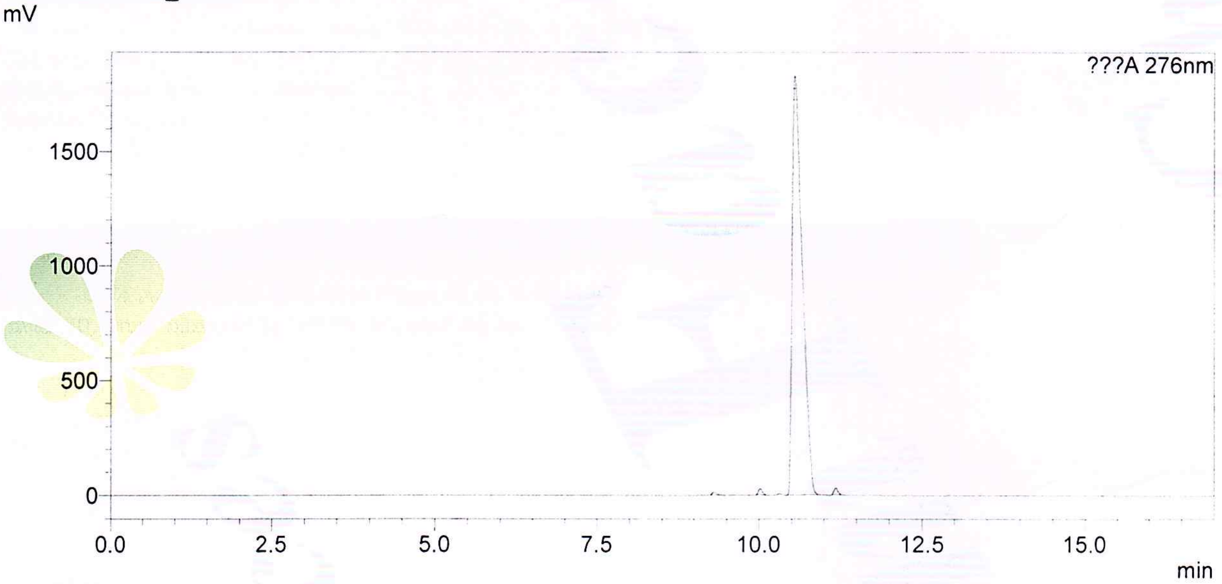

<Peak Table>

???A 276nm

Peak#	Ret. Time	Area	Height	Conc.	Unit	Area%
1	9.308	49736	11553	0.249		0.249
2	10.018	101655	26422	0.509		0.509
3	10.551	19704510	1829923	98.689		98.689
4	11.184	110305	28313	0.552		0.552
Total		19966206	1896210			100.000

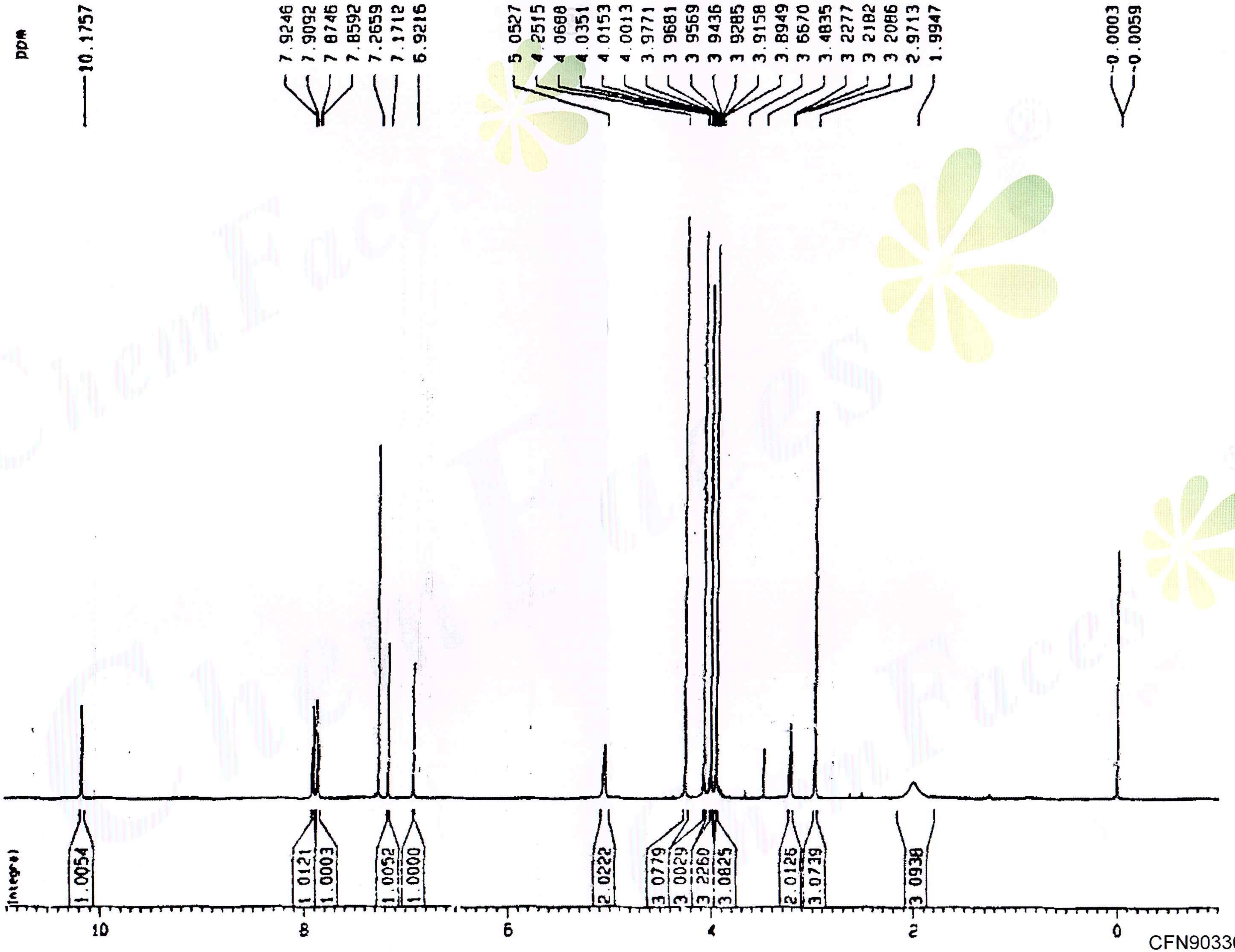

ppm
10.1757
7.9246
7.9092
7.8746
7.8592
7.2659
7.1712
6.9216
5.0527
4.2515
4.0688
4.0351
4.0153
4.0013
3.9771
3.9681
3.9569
3.9436
3.9285
3.9158
3.8949
3.6670
3.4835
3.2277
3.2182
3.2086
2.9713
1.9947
-0.0003
-0.0059
Integral
1.0054
1.0121
1.0003
1.0052
1.0000
2.0222
3.0779
3.0029
3.2260
3.0825
2.0126
3.0739
3.0938
10
8
6
4
2
0

CFN90330

Figure S2. HPLC and NMR profile of the compound.
